# Diet and Mental Health: Review of the Recent Updates on Molecular Mechanisms

**DOI:** 10.3390/antiox9040346

**Published:** 2020-04-23

**Authors:** Justyna Godos, Walter Currenti, Donato Angelino, Pedro Mena, Sabrina Castellano, Filippo Caraci, Fabio Galvano, Daniele Del Rio, Raffaele Ferri, Giuseppe Grosso

**Affiliations:** 1 Oasi Research Institute—IRCCS, 94018 Troina, Italy; carafil@hotmail.com (F.C.); rferri@oasi.en.it (R.F.); 2 Department of Biomedical and Biotechnological Sciences, University of Catania, 95123 Catania, Italy; currentiw@gmail.com (W.C.); fgalvano@unict.it (F.G.); giuseppe.grosso@unict.it (G.G.); 3 Faculty of Bioscience and Technology for Food, Agriculture and Environment, University of Teramo, 64100 Teramo, Italy; dangelino@unite.it; 4 Human Nutrition Unit, Department of Food and Drugs, University of Parma, 43125 Parma, Italy; pedromiguel.menaparreno@unipr.it; 5 Department of Educational Sciences, University of Catania, 95124 Catania, Italy; sabrina.castellano@unict.it; 6 Department of Drug Sciences, University of Catania, 95125 Catania, Italy; 7 School of Advanced Studies on Food and Nutrition, University of Parma, 43125 Parma, Italy; daniele.delrio@unipr.it; 8 Department of Veterinary Medicine, University of Parma, 43125 Parma, Italy

**Keywords:** oxidative stress, inflammation, polyphenols, mental health, diet, depression, sleep, brain, neurons, neuronal plasticity

## Abstract

Over the last decades, there has been a substantial increase in the prevalence of mental health disorders, including an increased prevalence of depression, anxiety, cognitive, and sleep disorders. Diet and its bioactive components have been recognized among the modifiable risk factors, possibly influencing their pathogenesis. This review aimed to summarize molecular mechanisms underlying the putative beneficial effects toward brain health of different dietary factors, such as micro- and macronutrient intake and habits, such as feeding time and circadian rhythm. The role of hormonal homeostasis in the context of glucose metabolism and adiponectin regulation and its impact on systemic and neuro-inflammation has also been considered and deepened. In addition, the effect of individual bioactive molecules exerting antioxidant activities and acting as anti-inflammatory agents, such as omega-3 fatty acids and polyphenols, considered beneficial for the central nervous system via modulation of adult neurogenesis, synaptic and neuronal plasticity, and microglia activation has been summarized. An overview of the regulation of the gut–brain axis and its effect on the modulation of systemic inflammation and oxidative stress has been provided. Finally, the impact of bioactive molecules on inflammation and oxidative stress and its association with brain health has been summarized.

## 1. Introduction

Over the last decade, mental health diseases have represented the major contributors to years of life lost due to disability in developed countries, while alarming trends have also been registered in developing ones [1]. Recent reports estimated that the global population living with depression is well over 300 million people and that anxiety disorders affect more than 260 million people (4.4% and 3.6% of the global population, respectively) [2]. Mental disorders account for 14% of worldwide Years Lived with Disability (YLD), with depressive disorders leading to a global total of over 50 million YLD and anxiety disorders to 24.6 million YLD in 2015 [3]. Furthermore, it is noteworthy that depression and anxiety are often associated, if not prodromic, to some of the other mental conditions and related to other non-communicable diseases, such as cardiovascular disease, dementia, and cancer [4]. Together with the aforementioned disorders, sleep disorders have been of interest as emerging conditions, mainly due to their possible link to other health conditions [5]. Recognizing early symptoms of mental disorders, identifying potential risk factors, and intervening to modify chronic exposure to them is of paramount importance to prevent the development of serious conditions fated to have a growing impact on the general population and future generations.

A growing body of literature has focused attention on potential risk factors for mental disorders, involving studies on (paleo)anthropology, human evolution, and biology, together with culture and environment [6]. On one side, we are witnesses of a fast evolution of human society, rise in technological advances, global industrialization and urbanization which has happened over the last 50 years; on the other side, very little genetic variation has occurred since the last thousands of years, leading to a potential evolutionary mismatch between our ancestral genome and the current environmental exposure [6]. The modern life led to the rise of non-communicable chronic diseases due to important changes in lifestyle factors, including, among others, adoption of unhealthy dietary patterns and sleeping habits [7]. Diet per se has been estimated to be the most important risk factor for non-communicable diseases in the modern era [8]. It has been calculated that dietary factors are responsible for 10.9 million deaths and 255 million Disability-Adjusted Life-Years [8]. The modern era is characterized by a “nutrition transition” process, characterized by a global shift from traditional dietary patterns toward so-called “Westernized” diets, rich in processed energy-dense food, refined sugars, trans-fatty acids, excessive sodium, and scarce consumption of plant-derived foods, accompanied by an unbalance between calorie intake and its expenditure through regular physical activity [6]. There is a large number of studies comprehensively considering the relation between diet and human health, showing not only an impact on cardio–metabolic diseases and certain cancers but also a potential role in affecting mental health disorders risk [9,10]. The urban environment may also lead to disruption of the circadian cycle due to continuous exposure to stimuli, such as light and acoustic pollution, busy working schedule, and societal challenges, which may play a role in anxiety and mood disorders, depression, cognitive, and sleep disorders [11].

For these reasons, there is a growing interest in the so-called “nutritional psychiatry”, which aims to describe and understand the relationship between dietary factors and mental health disorders. Over the last years, the number of studies investigating the role of individual dietary factors and overall dietary patterns toward depressive, anxiety, and sleep disorders provide an interesting body of evidence, although derived mostly from observational studies [12]. This review aims to provide an overview of the main molecular mechanisms hypothesized to explain the relationship between diet and mental health.

## 2. Role of Nutritional Components on the Brain: Micro- and Macro-Nutrients

Based on their specific metabolism and functions, somatic cells may have different requirements in terms of nutrients. Among these, brain cells are known to be surrounded by the blood–brain barrier layer, filtrating the passage of nutritional compounds, which may play a role in brain health.

**Figure 1:**
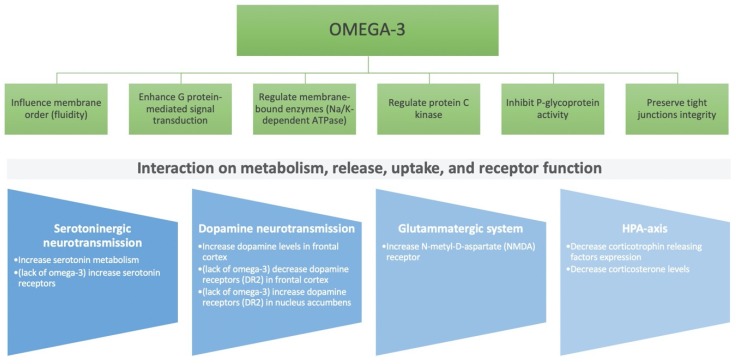
Neuroendocrine mechanisms of omega-3 polyunsaturated fatty acids effect on the central nervous system.

Among macronutrients, a large part of scientific literature has focused the interest on the role of fatty acids on mental health. The main polyunsaturated fatty acids (PUFAs) that exert effects on brain cells are docosahexaenoic acid (DHA) and eicosapentaenoic acid (EPA). DHA is a key structural component of membrane phospholipids in the brain, which is responsible for neuronal membrane integrity, fluidity, and functioning; EPA is a precursor of anti-inflammatory cytokines, an inhibitor of prostaglandins, thromboxanes and leukotrienes, that has been hypothesized to positively modulate both metabolic and immune processes, in contrast to omega-6 PUFA precursors of arachidonic acid (AA) demonstrated to be a pro-inflammatory mediator (Figure 1) [13]. Besides the action toward inflammation, omega-3 PUFA have been shown to modulate the neuroendocrine modulation of the serotoninergic and dopaminergic neuroendocrine transmission [14]. Furthermore, monounsaturated fatty acids (MUFA) have been shown to modulate brain activity and sleep behaviors, possibly by affecting glucose metabolism [15].

Proteins and amino acids derived from dietary intake can positively influence sleep quality and duration by modulating neurotransmitters and neuromodulators expression [16]. Among the most studied, tryptophan, an essential amino acid, is of major interest because it acts as an upstream precursor of bioactive metabolites related to sleep, including serotonin and melatonin [17]. Glycine, a non-essential amino acid, may exert a positive action toward sleep quality by reducing the core body temperature exerting an excitatory and inhibitory role on neurotransmission via *N*-methyl-d-aspartate-type glutamate receptors and glycine receptors, respectively [18]. l-ornithine, another non-essential, non-proteinogenic amino acid, may play a direct role in the central nervous system relieving stress and improving sleep and fatigue symptoms through reducing stress-induced activation of the hypothalamic–pituitary–adrenal (HPA) axis accompanied by a reduction in the serum corticosterone concentration and attenuating the stress response mediated by the GABA receptor [19].

Carbohydrates are needed as glucose is the main source of energy in the brain, while depressive and other mood disturbances may lead to their excessive consumption (“carbohydrate craving”) due to enhancement in brain serotonin synthesis [20]. Carbohydrates may have a different absorption rate and time, based on their food sources and composition, which in turn influence insulin response and blood glucose release. Consequently, the dietary glycemic load and index of foods have been associated with an acute inflammatory response: in fact, when a high-glycemic-index food source of carbohydrate is introduced, insulin secretion rapidly spikes for glucose cell internalization through the different GLUT transporters, to maintain blood glucose homeostasis. Insulin also stimulates the uptake of branched-chain amino acids into skeletal muscle, but not the uptake of tryptophan, which is largely bound to plasma albumin. This makes tryptophan preferentially available to the large neutral amino acid transporter at the blood–brain barrier, with the consequent conversion to serotonin [21]. However, some studies have shown that excessive chronic consumption of available carbohydrates was associated with a poorer hippocampal-related memory; this relationship might be mediated by increased neuro-inflammation in the hippocampus, typically present on high sugar/low-fat conditions [21]. In contrast, a high intake of complex carbohydrates as fiber from vegetables, fruits, legumes, and whole grains might have a beneficial role in mental health. In fact, this type of carbohydrate is not digestible by human amylases and is broken down by the gut bacteria leading to the release of short-chain fatty acids (SCFAs), including acetate, propionate, and butyrate. These molecules have shown anti-inflammatory effects that can also be transmitted to the brain via pathways involving direct central nervous system signaling (see paragraph gut–brain axis) and the immune system activation (see paragraph Inflammation) [22,23].

Among micronutrients, B-group vitamins may modulate cognitive performance, preserve memory during aging, and improve cerebral and cognitive functions in the elderly by reducing homocysteine levels [24]. Moreover, niacin may be a potential agent used to support the treatment of schizophrenia [25]. Vitamin D may be involved in the prevention of neurodegenerative disorders; alpha-tocopherol, one of the compounds of the vitamin E group, is involved in nervous membranes protection from oxidative damage [26]. Among minerals, manganese, zinc, and copper participate in enzymatic mechanisms protecting against oxidative stress; iron plays a role for oxygenation, energy production, and neurotransmitters and myelin synthesis in the cerebral parenchyma; and calcium, potassium, and magnesium modulate sleep through the proper functioning of ion channels [27].

Bioactive compounds have also been widely investigated for their putative role in the prevention of cognitive decline and amelioration of the cognitive functions. Among the studies, the MIND Diet Study was among the pioneering studies evidencing that the frequent consumption of berries, nuts, green leafy vegetables, and whole-grain cereals, all particularly rich in polyphenols, was associated with a significant delay in lowering of cognitive decline [28,29]. Other than epidemiological data, the PREDIMED study evidenced a positive association between the higher urinary polyphenol content quartile and the improvement in verbal language tests [30]. An exhaustive review by Schaffer et al. 2012 [31] elucidated that polyphenols are able to alter brain function at three levels: (i) outside the central nervous system, i.e., by improving cerebral blood flow; (ii) at blood brain barrier (BBB) level, i.e., by modulating transporter activities, such as multi-drug resistant protein-dependent, which regulates influx and efflux of various biomolecules; (iii) inside the central nervous system, by directly modifying the activity of neurons and glial cells.

Glucosinolates, sulfur compounds mainly present in *Brassicacea* family plants, are biologically inactive compounds that are converted, after vegetable chewing, into different bioactive compounds, such as isothiocyanates or indoles. A recent review summarized the main activities of these compounds at neuronal levels, mainly through the positive modulation of the Nuclear factor erythroid 2 related factor (Nrf2) antioxidant pathway and the reduced synthesis of pro-inflammatory cytokines [32].

Carotenoids, lipophilic compounds mainly present in orange and red vegetables, but also in *Brassicaceae,* have also been considered for cognitive function effects in large cohorts, such as NHANES. In this cohort, the highest quartile of intake of lutein and zeaxanthin is correlated with the best improvement in cognitive tests [33].

## 3. Feeding Time, Circadian Rhythm, and Hormonal Homeostasis

The circadian cycle regulates and coordinates many biological processes, such as the sleep–wake cycle, hormone secretion, glucose homeostasis, and thermogenesis. The periodicity of behavioral and metabolic processes is determined by circadian rhythms that are 24-hour cycles [34]. Several environmental and lifestyle factors have been proven to be stimuli for the circadian cycle, including hormones, physical activity, nutrients and their patterns, feeding and fasting state, sleep–wake state, and temperature [35]. As the effect of circadian rhythm on metabolic processes and energy balance is bidirectional, any detrimental effects of the stimuli may cause energy imbalance and, thus, lead to a higher risk of age-related diseases.

The fasting–refeeding physiology is based on the 24-hour cycle capacity to acquire food when it is available and to store and utilize these resources during the rest of the day without compromising fitness and vitality [36]. According to recent evidence, the fasting period is believed to serve as a time for repair and renewal of the organism components, as the theory of the physiology of fasting states that certain biochemical processes are triggered once stored resources are being utilized, and not during the feeding period [37]. One of the possible mechanisms underlying the relationship between circadian rhythm, sleep, and metabolism, includes adiponectin, a hormone involved in glucose metabolism. As it appears, the relationship between adiponectin and the circadian system is bidirectional, and its expression has been proven to be circadian periodic [38]. Adiponectin levels increase significantly in response to intermittent fasting, and higher levels of adiponectin have been inversely associated with risk of cardiovascular diseases [38]; interestingly, recent data suggest that sleep restriction may decrease levels of adiponectin in healthy individuals and, thus, contribute to the risk of cardiovascular diseases [39]. Finally, lower peripheral adiponectin levels have been associated with anxiety, mood, and stress-related affective disorders [40], suggesting a pleiotropic effect of adiponectin toward mental health.

Much attention has also been paid to the brain-derived neurotrophic factor (BDNF), which is a fundamental neurotrophin regulating brain functions as a neurotransmitter modulator, modulating neuronal survival and growth, and participating in neuronal plasticity [41]. Additionally, it has been shown to exert a key role in glucose and energy metabolism since receptors for both BDNF and insulin are coupled to PI3-kinase/Akt and MAP kinase intracellular signaling pathways [41]. Experimental studies have determined that intermittent fasting increases BDNF expression in several regions of the brain, and BDNF, at least in part, mediates intermittent fasting-induced neurogenesis, promoting synaptic plasticity and increasing neuronal resistance to injury and disease. It may also mediate behavioral and metabolic responses to fasting, including regulation of appetite, peripheral glucose metabolism, and autonomic control of the cardiovascular and gastrointestinal systems [42]. A decreased serum level of BDNF has been associated with insomnia and sleep deprivation, and it has been demonstrated that subjects suffering from current symptoms of depression and insomnia exhibited significantly decreased serum BDNF levels [43,44]. Significant fluctuations of gut microbiota during the day–night shift may result in time-of-day-specific taxonomic configurations related not only to rhythmic food intake and dietary structure but also to the biological clock, suggesting an interaction between microorganisms and circadian genes as well as emotion and physiological stress [45].

Finally, bioactive compounds have also been shown to play a role in the regulation of the circadian rhythm and, in turn, in the modulation of obesity-related outcomes. Epigallocatehin-3-gallate (EGCG), one of the most abundant flavan-3-ol in green tea, has been evaluated for its role in the modulation of circadian clock genes, such as *Clock*, *Bmal1*, *Cry1*, which may then regulate genes, such as *Sirt1* and *PGC1α*, controlling lipid metabolism in adipose tissue [46]. Not only circadian rhythm but also circannual rhythms have been found to be modulated by polyphenols. It has been shown that the consumption of polyphenol-rich fruit and vegetables in their specific season or out of season may alter the photoperiod-dependent effects, leading to different regulation of genes responsible for the fatty acid transport and lipolysis in skeletal muscles [47] Similarly, the consumption of seasonal fruit and vegetables, possibly through their polyphenol content, is able to influence leptin system depending on the photoperiod in which they are consumed [48].

## 4. Gut–Brain Axis

In healthy adults, the composition of the intestinal microbiota is generally stable over time, with the bacterial phyla, such as Firmicutes (including *Lactobacillus*, *Clostridium*, and *Enterococcus* genus) and Bacteroidetes (i.e., *Bacteroides* genus), dominating gut microbiome [49]. Changes in the gut microbiome characterized by excessive presence of facultative anaerobes (*Escherichia coli*), pro-inflammatory *Ruminococcus*, or nonbacterial microbes may lead to pathogenic conditions [50]. Thus, the diversity and equilibrium of the gut microbiota strains are important indices for the overall body health [51]. Moreover, type, quality, and origin of food shape the gut microbiota profile and affect its composition and function [52]. Higher intake of fiber [53], pre- and probiotics [54] have been shown to modulate the gut microbiota. Healthy dietary patterns, such as the Mediterranean diets and other plant-rich diets, have been related to increased diversity of the microbiota [55]. Albeit rather preliminary, some studies have shown a possible correlation between gut microbiota composition (i.e., a modification in the *Firmicutes*/*Bacteroidetes*/*Clostridium* ratio) and depressive state and response to chronic stress [56].

**Figure 2:**
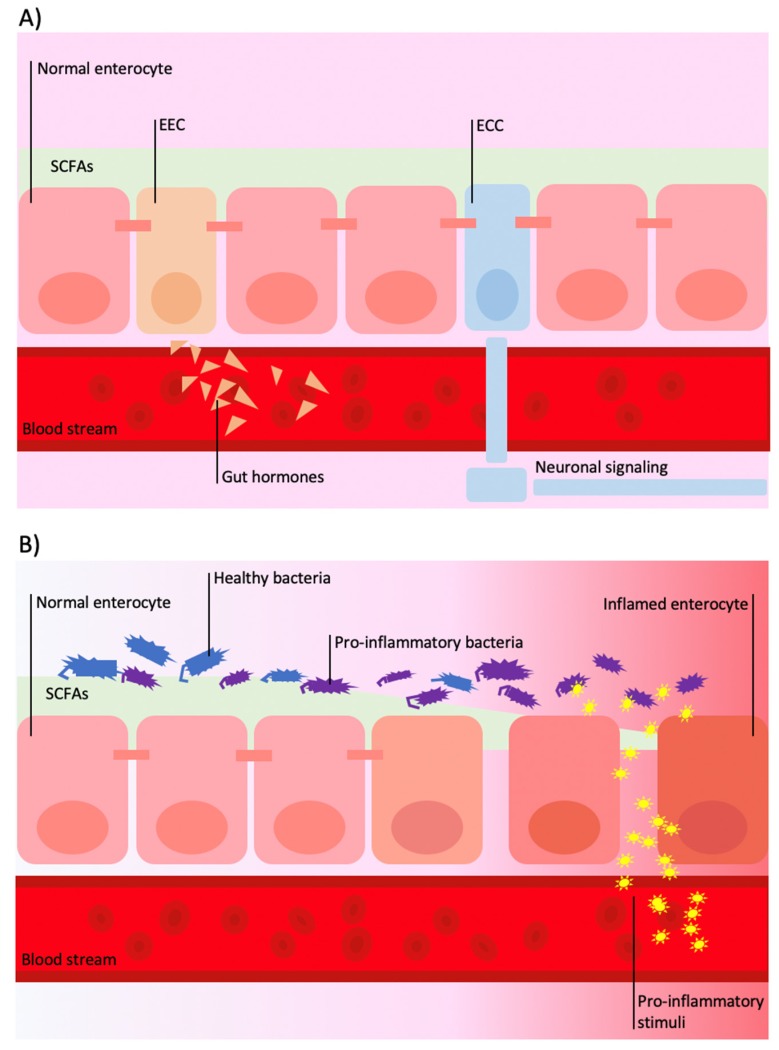
Schematic illustration of gut–brain communication through systemic involvement. (**A**) In physiologic conditions, the gut is able to transmit signals at systemic level by activation of enteroendocrine cells (EECs) and enterochromaffins cells (ECCs): EECs are responsible for secretion of various circulating hormones (i.e., glucagon-like peptides (GLPs), peptide tyrosine tyrosine (PYY), oxyntomodulin); ECCs are responsible for direct neuronal signaling through vagal or afferent nerve fibers by controlling serotonin release. (**B**) Modification of gut microbiota, such as a decrease in commensal Bifidobacterium and Lactobacillus strains and increase in Bacteroides/Prevotella species, may lead to a reduction of short-chain fatty acids (SCFAs) including acetate, propionate, and butyrate and hyper-secretion of lipopolysaccharides (LPS) that bind to circulating monocyte and macrophages, which, in turn, stimulate the secretion of pro-cytokines (TNF-a, IL-1, IL-6).

Current research emphasizes the interexchange of signals influenced by the gut microbiota that are detected and transduced in information from the gut to the nervous system involving neural, endocrine, and inflammatory mechanisms [57]. Gut microbiota has been shown to directly affect neurotransmitter metabolism with implications for enteric and central nervous system function through the production of molecules, such as SCFAs, secondary bile acids, and tryptophan metabolites [58], together with folate and GABA [59]. The signal can be propagated by interaction with enteroendocrine cells (EECs) and enterochromaffins cells (ECCs), which are able to induce central responses (i.e., by controlling serotonin release) via long-distance neural signaling by vagal or afferent nerve fibers that extend into intestinal villi (Figure 2A) [60]. 

The intestinal microbiota plays a role in gut peptides’ modulation, which, in turn, are part of the complex pathway characterizing the gut–brain axis [61]. The neuropeptide Y, which is among the most abundant peptides in the brain (including the nucleus of the solitary tract, hypothalamus, and amygdala) and highly regulated by peripheral signaling, is able to regulate the release of GABA [62]. Another mechanism may rely on glucagon-like peptide-1, an incretin hormone, which is involved in the modulation of the HPA axis and overall response to stress as well as playing a role in lowering postprandial blood glucose via augmentation of glucose-dependent insulin release and inhibition of glucagon secretion [63]. Cholecystokinin (CKK) is a peptide able to control gastric emptying, gallbladder contraction, pancreatic enzyme release, and suppression of appetite. At the central nervous system level, it has been demonstrated to play a role in anxiety-like behavior through the activation of the CCK2 receptors in limbic regions [64]. Serum ghrelin, which is known for its adipogenic effects and for playing a role in response to stress (i.e., triggering motivation for rewards), has been shown to be associated with modification of certain gut bacteria strains, such as negatively correlated with the commensal *Bifidobacterium* and *Lactobacillus* strains, and directly with *Bacteroides*/*Prevotella* species [65]. Corticotropin-releasing factor (CRF) plays a key role in response to stress mediating the neural control of adrenocorticotropic hormone (ACTH) release from pituitary corticotrophs, which in turn regulate cortisol secretion acutely but may lead to the development of stress-related disorders (i.e., anxiety and depression) when exposed to chronic stress [66]. The CRF system also influences some functions within the gastrointestinal system, including gut motility and permeability [67]. Interestingly, animal studies showed that increased CRF might be associated with alteration in the intestinal microbial community (i.e., reduction in Lactobacillus), as well, an opposite relation has been found between alteration of the CRF signaling (i.e., CRF-mediated activation of the HPA axis) and changes in the gut microbiota [68].

Among one of the most interesting links between brain and gut microbiota is the rearrangement of the (poly)phenols structure by the latter [69], resulting in the release into the bloodstream of smaller compounds, putatively active at the nervous system level. Among these, the chemical rearrangement of flavan-3-ols, one of the most common flavonoid subclasses, has been extensively studied [70]. The main flavan-3-ol colonic metabolites, namely phenyl-γ-valerolactones, have been recently found to be able to cross the blood–brain barrier and be available to the neuronal cell [71], where they may effectively interfere with amyloid-β oligomers assembly, representing a potential novel pharmacological tool to prevent Aβ aggregation and Aβ-induced neurodegeneration in Alzheimer’s disease (AD) [72]. Additionally, some more other small colonic metabolites are the object of similar studies, as they have been able to exert anti-inflammatory activity at the neuronal level [73]. In terms of human intervention studies, in a recent trial, an acute supplementation of a grape and blueberry polyphenol-rich extract was found to improve cognitive performance with a major impact on specific cognitive functions, such as working memory and attention [74].

Among indirect mechanisms, an impaired intestinal microbiota characterized by a reduced number of different species and a prevalence of detrimental bacterial species over others (so-called “*dysbiosis*”), contributes to increased permeability of the intestinal mucosa (“leaky gut”) (Figure 2B). This clinical condition facilitates the passage of some bacterial components as lipopolysaccharides (LPS) that bind to circulating monocyte and macrophages, which in turn stimulate the secretion of cytokines (TNF-a, IL-1, IL-6) causing an increase in the inflammatory state [21]. SCFAs may exert anti-inflammatory action by binding to G-protein receptors found in multiple cells, including nerve fibers, EECs, glial cells in the brain, and adipocytes, which suppress a neuroinflammatory response, i.e., against the LPS inflammatory responses in microglia [75]. Moreover, there is evidence of a direct anti-inflammatory action through the promotion of microglial activation [76]. Furthermore, gut neurotransmitters, such as serotonin, have been shown to exert both pro-inflammatory and anti-inflammatory functions, thus playing a role in the modulation of immune and inflammatory responses [77].

## 5. Inflammation and Oxidative Stress

Low-grade inflammation, characterized by the presence of pro-inflammatory cytokines in the bloodstream while incurring no clinical symptoms, and oxidative stress parameters, and antioxidant capacity have been reported to potentially play a role in several non-communicable diseases, including mental disorders [78]. Cytokines are soluble intercellular signaling molecules that are involved in the pathophysiology of several mental disorders through affecting neurotransmitter synthesis, release, and reuptake [79], as well as regulating BDNF expression [80]. In regard to mental disorders, there is a growing body of research investigating the possible cross-talk between pathological alterations in sleep patterns [81], depression [82], and anxiety disorders [83] and inflammation-related diseases that involve increased inflammatory cytokines release. On the other hand, different studies have also shown that a deficit of anti-inflammatory cytokines, such as transforming growth factor-β1 (TGF-β1), can contribute to the pathogenesis of depressive disorders [84]. Interestingly, TGF-β1 plays a key role as a regulator of gut microbiota [85], and its deficit plays a central role in the pathophysiology of cognitive deficits both in depression and AD [84]. Gut dysbiosis-dependent fluctuations of butyrate can interfere with TGF-β1 synthesis and release from intestinal epithelial cells [86].

It is hypothesized that sleep disruption may have an effect on the inflammatory state, activating microglia in the central nervous system [21], reducing synaptic plasticity and hippocampal neurogenesis, altering neurotransmitters production, and gene transcription determining epigenetic changes that in turn lead to short-term and long-lasting imbalances of neuronal function and behavior [21]. Similarly, depression and anxiety disorders have been associated with higher inflammatory status and disruption in brain functionality and may, therefore, be linked to sleep disorders [83]. Some recent studies have shown a bidirectional link between mental health, inflammation, and oxidative stress. Poor sleep quality, depression, and anxiety may be associated with higher levels of IL-6 and CRP [87]. Even dietary patterns and nutrients intake can influence the expression of inflammatory biomarkers, which in turn have an effect on neuroinflammation. It has been hypothesized that several dietary factors may have an effect on systemic inflammation through pro- or anti-inflammatory cytokine secretion and regulation of the nuclear factor kappa-light-chain-enhancer of the activated B cells (NF-κB) signaling pathway [88]. It has been demonstrated that plant-based food, such as fruits, vegetables, legumes, and whole grains, exert neuroprotective and anti-inflammatory effects, thanks to the high content of vitamins and polyphenols [21]. Similarly, MUFA from extra virgin olive oil and certain PUFA as omega-3 from fish exert anti-inflammatory effects, which, in turn, improve cognitive function [21]. At the opposite, foods rich in calories, added sugars, hydrogenated fats, and preservatives may determine the secretion of pro-inflammatory cytokines and, therefore, worsen both inflammatory state and cognitive function [21]. Similarly, high glycemic load meals and consumption of processed meat products have been associated with the production of inflammatory biomarkers [89,90]. Finally, the previously mentioned phenyl-γ-valerolactones, originating by gut microbiota transformation of flavan-3-ols, may exert their neuroprotective activity in secondary prevention strategies for AD also through an anti-inflammatory mechanism, having been shown to be able to reduce glial over-activation in amyloid-β oligomers-treated mice [72].

## 6. Conclusions

Most of the mechanisms hypothesized to explain the potential relation between diet and mental health are strictly connected. No univocal dietary component should be considered as a determinant for the improvement in mental health outcomes, while a general group of features of the whole dietary and eating habits seems to better describe the potential beneficial effects of diet with a synergistic interaction between different nutrients in the prevention of mental health disorders.

## References

[1] Whiteford H.A., Degenhardt L., Rehm J., Baxter A.J., Ferrari A.J., Erskine H.E., Charlson F.J., Norman R.E., Flaxman A.D., Johns N. (2013). Global burden of disease attributable to mental and substance use disorders: Findings from the Global Burden of Disease Study 2010. Lancet.

[2] Friedrich M.J. (2017). Depression is the leading cause of disability around the world. JAMA.

[3] GBD Disease and Injury Incidence and Prevalence Collaborators (2018). Global, regional, and national incidence, prevalence, and years lived with disability for 354 diseases and injuries for 195 countries and territories, 1990–2017: A systematic analysis for the Global Burden of Disease Study 2017. Lancet.

[4] Clarke D.M., Currie K.C. (2009). Depression, anxiety and their relationship with chronic diseases: A review of the epidemiology, risk and treatment evidence. Med. J. Aust..

[5] Frank S., Gonzalez K., Lee-Ang L., Young M.C., Tamez M., Mattei J. (2017). Diet and sleep physiology: Public health and clinical implications. Front. Neurol..

[6] Logan A.C., Jacka F.N. (2014). Nutritional psychiatry research: An emerging discipline and its intersection with global urbanization, environmental challenges and the evolutionary mismatch. J. Physiol. Anthropol..

[7] Branca F., Lartey A., Oenema S., Aguayo V., Stordalen G.A., Richardson R., Arvelo M., Afshin A. (2019). Transforming the food system to fight non-communicable diseases. BMJ.

[8] GBD Risk Factor Collaborators (2018). Global, regional, and national comparative risk assessment of 84 behavioural, environmental and occupational, and metabolic risks or clusters of risks for 195 countries and territories, 1990–2017: A systematic analysis for the Global Burden of Disease Study 2017. Lancet.

[9] GBD Diet Collaborators (2019). Health effects of dietary risks in 195 countries, 1990–2017: A systematic analysis for the Global Burden of Disease Study 2017. Lancet.

[10] Dominguez L.J., Barbagallo M., Munoz-Garcia M., Godos J., Martinez-Gonzalez M.A. (2019). Dietary patterns and cognitive decline: Key features for prevention. Curr. Pharm. Des..

[11] Pot G.K. (2018). Sleep and dietary habits in the urban environment: The role of chrono-nutrition. Proc. Nutr. Soc..

[12] Lassale C., Batty G.D., Baghdadli A., Jacka F., Sanchez-Villegas A., Kivimaki M., Akbaraly T. (2019). Healthy dietary indices and risk of depressive outcomes: A systematic review and meta-analysis of observational studies. Mol. Psychiatry.

[13] Su K.P. (2008). Mind-Body interface: The role of n-3 fatty acids in psychoneuroimmunology, somatic presentation, and medical illness comorbidity of depression. Asia Pac. J. Clin. Nutr..

[14] Grosso G., Galvano F., Marventano S., Malaguarnera M., Bucolo C., Drago F., Caraci F. (2014). Omega-3 fatty acids and depression: Scientific evidence and biological mechanisms. Oxidative Med. Cell. Longev..

[15] Sartorius T., Ketterer C., Kullmann S., Balzer M., Rotermund C., Binder S., Hallschmid M., Machann J., Schick F., Somoza V. (2012). Monounsaturated fatty acids prevent the aversive effects of obesity on locomotion, brain activity, and sleep behavior. Diabetes.

[16] Glenn J.M., Madero E.N., Bott N.T. (2019). Dietary protein and amino acid intake: Links to the maintenance of cognitive health. Nutrients.

[17] Friedman M. (2018). Analysis, nutrition, and health benefits of tryptophan. Int. J. Tryptophan Res..

[18] Bannai M., Kawai N. (2012). New therapeutic strategy for amino acid medicine: Glycine improves the quality of sleep. J. Pharmacol. Sci..

[19] Kurata K., Nagasawa M., Tomonaga S., Aoki M., Akiduki S., Morishita K., Denbow D.M., Furuse M. (2012). Orally administered L-ornithine reduces restraint stress-induced activation of the hypothalamic-pituitary-adrenal axis in mice. Neurosci. Lett..

[20] Wurtman J., Wurtman R. (2018). The trajectory from mood to obesity. Curr. Obes. Rep..

[21] Irwin M.R., Olmstead R., Carroll J.E. (2016). Sleep disturbance, sleep duration, and inflammation: A systematic review and meta-analysis of cohort studies and experimental sleep deprivation. Biol. Psychiatry.

[22] Ceppa F., Mancini A., Tuohy K. (2019). Current evidence linking diet to gut microbiota and brain development and function. Int. J. Food Sci. Nutr..

[23] Salvucci E. (2019). The human-microbiome superorganism and its modulation to restore health. Int. J. Food Sci. Nutr..

[24] Smith A.D., Refsum H. (2016). Homocysteine, B vitamins, and cognitive impairment. Annu. Rev. Nutr..

[25] Hoffer A., Prousky J. (2008). Successful treatment of schizophrenia requires optimal daily doses of vitamin B3. Altern. Med. Rev..

[26] Bourre J.M. (2006). Effects of nutrients (in food) on the structure and function of the nervous system: Update on dietary requirements for brain. Part 1: Micronutrients. J. Nutr. Health Aging.

[27] Zeng Y., Yang J., Du J., Pu X., Yang X., Yang S., Yang T. (2014). Strategies of functional foods promote sleep in human being. Curr. Signal Transduct. Ther..

[28] Morris M.C., Tangney C.C., Wang Y., Sacks F.M., Barnes L.L., Bennett D.A., Aggarwal N.T. (2015). MIND diet slows cognitive decline with aging. Alzheimers Dement..

[29] Morris M.C., Tangney C.C., Wang Y., Sacks F.M., Bennett D.A., Aggarwal N.T. (2015). MIND diet associated with reduced incidence of Alzheimer’s disease. Alzheimers Dement..

[30] Valls-Pedret C., Lamuela-Raventos R.M., Medina-Remon A., Quintana M., Corella D., Pinto X., Martinez-Gonzalez M.A., Estruch R., Ros E. (2012). Polyphenol-Rich foods in the Mediterranean diet are associated with better cognitive function in elderly subjects at high cardiovascular risk. J. Alzheimers Dis..

[31] Schaffer S., Halliwell B. (2012). Do polyphenols enter the brain and does it matter? Some theoretical and practical considerations. Genes Nutr..

[32] Jaafaru M.S., Abd Karim N.A., Enas M.E., Rollin P., Mazzon E., Abdull Razis A.F. (2018). Protective effect of glucosinolates hydrolytic products in neurodegenerative diseases (NDDs). Nutrients.

[33] Christensen K., Gleason C.E., Mares J.A. (2018). Dietary carotenoids and cognitive function among US adults, NHANES 2011–2014. Nutr. Neurosci..

[34] Longo V.D., Mattson M.P. (2014). Fasting: Molecular mechanisms and clinical applications. Cell Metab..

[35] Van Gelder R.N., Buhr E.D. (2016). Ocular photoreception for circadian rhythm entrainment in mammals. Annu. Rev. Vis. Sci..

[36] Serin Y., Acar Tek N. (2019). Effect of circadian rhythm on metabolic processes and the regulation of energy balance. Ann. Nutr. Metab..

[37] Longo V.D., Panda S. (2016). Fasting, circadian rhythms, and time-restricted feeding in healthy lifespan. Cell Metab..

[38] Cornelissen G. (2018). Metabolic syndrome, adiponectin, sleep, and the circadian system. EBioMedicine.

[39] Simpson N.S., Banks S., Arroyo S., Dinges D.F. (2010). Effects of sleep restriction on adiponectin levels in healthy men and women. Physiol. Behav..

[40] Vuong E., Nothling J., Lombard C., Jewkes R., Peer N., Abrahams N., Seedat S. (2020). Peripheral adiponectin levels in anxiety, mood, trauma-and stressor-related disorders: A systematic review and meta-analysis. J. Affect. Disord..

[41] Monteiro B.C., Monteiro S., Candida M., Adler N., Paes F., Rocha N., Nardi A.E., Murillo-Rodriguez E., Machado S. (2017). Relationship between brain-derived neurotrofic factor (Bdnf) and sleep on depression: A critical review. Clin. Pract. Epidemiol. Ment. Health.

[42] Mattson M.P. (2012). Energy intake and exercise as determinants of brain health and vulnerability to injury and disease. Cell Metab..

[43] Schmitt K., Holsboer-Trachsler E., Eckert A. (2016). BDNF in sleep, insomnia, and sleep deprivation. Ann. Med..

[44] Castren E., Rantamaki T. (2010). The role of BDNF and its receptors in depression and antidepressant drug action: Reactivation of developmental plasticity. Dev. Neurobiol..

[45] Li Y., Hao Y., Fan F., Zhang B. (2018). The role of microbiome in insomnia, circadian disturbance and depression. Front. Psychiatry.

[46] Arola-Arnal A., Cruz-Carrion A., Torres-Fuentes C., Avila-Roman J., Aragones G., Mulero M., Bravo F.I., Muguerza B., Arola L., Suarez M. (2019). Chrononutrition and polyphenols: Roles and diseases. Nutrients.

[47] Marine-Casado R., Domenech-Coca C., Del Bas J.M., Blade C., Caimari A., Arola L. (2019). Cherry consumption out of season alters lipid and glucose homeostasis in normoweight and cafeteria-fed obese Fischer 344 rats. J. Nutr. Biochem..

[48] Ibars M., Aragones G., Ardid-Ruiz A., Gibert-Ramos A., Arola-Arnal A., Suarez M., Blade C. (2018). Seasonal consumption of polyphenol-rich fruits affects the hypothalamic leptin signaling system in a photoperiod-dependent mode. Sci. Rep..

[49] Cenit M.C., Sanz Y., Codoner-Franch P. (2017). Influence of gut microbiota on neuropsychiatric disorders. World J. Gastroenterol..

[50] Hills R.D., Pontefract B.A., Mishcon H.R., Black C.A., Sutton S.C., Theberge C.R. (2019). Gut microbiome: Profound implications for diet and disease. Nutrients.

[51] Fava F., Rizzetto L., Tuohy K.M. (2018). Gut microbiota and health: Connecting actors across the metabolic system. Proc. Nutr. Soc..

[52] Dawson S.L., Dash S.R., Jacka F.N. (2016). The importance of diet and gut health to the treatment and prevention of mental disorders. Int. Rev. Neurobiol..

[53] Makki K., Deehan E.C., Walter J., Backhed F. (2018). The impact of dietary fiber on gut microbiota in host health and disease. Cell Host Microbe.

[54] Houghton D., Hardy T., Stewart C., Errington L., Day C.P., Trenell M.I., Avery L. (2018). Systematic review assessing the effectiveness of dietary intervention on gut microbiota in adults with type 2 diabetes. Diabetologia.

[55] St-Onge M.P., Zuraikat F.M. (2019). Reciprocal roles of sleep and diet in cardiovascular health: A review of recent evidence and a potential mechanism. Curr. Atheroscler. Rep..

[56] Dash S., Clarke G., Berk M., Jacka F.N. (2015). The gut microbiome and diet in psychiatry: Focus on depression. Curr. Opin. Psychiatry.

[57] Osadchiy V., Martin C.R., Mayer E.A. (2019). The gut-brain axis and the microbiome: Mechanisms and clinical implications. Clin. Gastroenterol. Hepatol..

[58] O’Mahony S.M., Clarke G., Borre Y.E., Dinan T.G., Cryan J.F. (2015). Serotonin, tryptophan metabolism and the brain-gut-microbiome axis. Behav. Brain Res..

[59] Tuohy K.M., Venuti P., Cuva S., Furlanello C., Gasperotti M., Mancini A., Ceppa F., Cavalieri D., de Filippo C., Vrhovsek U. (2015). Diet and the Gut Microbiota—How the Gut: Brain Axis Impacts on Autism.

[60] Gershon M.D. (2013). 5-Hydroxytryptamine (serotonin) in the gastrointestinal tract. Curr. Opin. Endocrinol. Diabetes Obes..

[61] Lach G., Schellekens H., Dinan T.G., Cryan J.F. (2018). Anxiety, depression, and the microbiome: A role for gut peptides. Neurotherapeutics.

[62] Martin C.R., Osadchiy V., Kalani A., Mayer E.A. (2018). The brain-gut-microbiome axis. Cell. Mol. Gastroenterol. Hepatol..

[63] Zietek T., Rath E. (2016). Inflammation meets metabolic disease: Gut feeling mediated by GLP-1. Front. Immunol..

[64] Ballaz S. (2017). The unappreciated roles of the cholecystokinin receptor CCK(1) in brain functioning. Rev. Neurosci..

[65] Morris L.S., Voon V., Leggio L. (2018). Stress, motivation, and the gut-brain axis: A focus on the ghrelin system and alcohol use disorder. Alcohol. Clin. Exp. Res..

[66] Fox J.H., Lowry C.A. (2013). Corticotropin-Releasing factor-related peptides, serotonergic systems, and emotional behavior. Front. Neurosci..

[67] Galley J.D., Bailey M.T. (2014). Impact of stressor exposure on the interplay between commensal microbiota and host inflammation. Gut Microbes.

[68] Chatoo M., Li Y., Ma Z., Coote J., Du J., Chen X. (2018). Involvement of corticotropin-releasing factor and receptors in immune cells in irritable bowel syndrome. Front. Endocrinol..

[69] Del Rio D., Rodriguez-Mateos A., Spencer J.P., Tognolini M., Borges G., Crozier A. (2013). Dietary (poly)phenolics in human health: Structures, bioavailability, and evidence of protective effects against chronic diseases. Antioxid. Redox Signal..

[70] Mena P., Bresciani L., Brindani N., Ludwig I.A., Pereira-Caro G., Angelino D., Llorach R., Calani L., Brighenti F., Clifford M.N. (2019). Phenyl-γ-valerolactones and phenylvaleric acids, the main colonic metabolites of flavan-3-ols: Synthesis, analysis, bioavailability, and bioactivity. Nat. Prod. Rep..

[71] Angelino D., Carregosa D., Domenech-Coca C., Savi M., Figueira I., Brindani N., Jang S., Lakshman S., Molokin A., Urban J.F. (2019). 5-(Hydroxyphenyl)-γ-valerolactone-sulfate, a key microbial metabolite of Flavan-3-ols, is able to reach the brain: Evidence from different in silico, In Vitro and In Vivo experimental models. Nutrients.

[72] Ruotolo R., Minato I., La Vitola P., Artioli L., Curti C., Franceschi V., Brindani N., Amidani D., Colombo L., Salmona M. (2020). Flavonoid-Derived human phenyl-gamma-valerolactone metabolites selectively detoxify amyloid-β oligomers and prevent memory impairment in a mouse model of Alzheimer’s disease. Mol. Nutr. Food Res..

[73] Carregosa D., Carecho R., Figueira I., Santos C.N. (2020). Low-Molecular weight metabolites from polyphenols as effectors for attenuating neuroinflammation. J. Agric. Food Chem..

[74] Philip P., Sagaspe P., Taillard J., Mandon C., Constans J., Pourtau L., Pouchieu C., Angelino D., Mena P., Martini D. (2019). Acute intake of a grape and blueberry polyphenol-rich extract ameliorates cognitive performance in healthy young adults during a sustained cognitive effort. Antioxidants.

[75] Sherwin E., Sandhu K.V., Dinan T.G., Cryan J.F. (2016). May the force be with you: The light and dark sides of the microbiota-gut-brain axis in neuropsychiatry. CNS Drugs.

[76] Kaczmarczyk M.M., Miller M.J., Freund G.G. (2012). The health benefits of dietary fiber: Beyond the usual suspects of type 2 diabetes mellitus, cardiovascular disease and colon cancer. Metabolism.

[77] Khan W.I., Ghia J.E. (2010). Gut hormones: Emerging role in immune activation and inflammation. Clin. Exp. Immunol..

[78] Cunningham C. (2013). Microglia and neurodegeneration: The role of systemic inflammation. Glia.

[79] Alam R., Abdolmaleky H.M., Zhou J.R. (2017). Microbiome, inflammation, epigenetic alterations, and mental diseases. Am. J. Med. Genet. B Neuropsychiatr. Genet..

[80] Zhang J.C., Yao W., Hashimoto K. (2016). Brain-Derived neurotrophic factor (BDNF)-TrkB signaling in inflammation-related depression and potential therapeutic targets. Curr. Neuropharmacol..

[81] Clark I.A., Vissel B. (2014). Inflammation-Sleep interface in brain disease: TNF, insulin, orexin. J. Neuroinflamm..

[82] Lee C.H., Giuliani F. (2019). The role of inflammation in depression and fatigue. Front. Immunol..

[83] Salim S., Chugh G., Asghar M. (2012). Inflammation in anxiety. Adv. Protein Chem. Struct. Biol..

[84] Caraci F., Spampinato S.F., Morgese M.G., Tascedda F., Salluzzo M.G., Giambirtone M.C., Caruso G., Munafo A., Torrisi S.A., Leggio G.M. (2018). Neurobiological links between depression and AD: The role of TGF-β1 signaling as a new pharmacological target. Pharmacol. Res..

[85] Bauche D., Marie J.C. (2017). Transforming growth factor β: A master regulator of the gut microbiota and immune cell interactions. Clin. Transl. Immunol..

[86] Martin-Gallausiaux C., Beguet-Crespel F., Marinelli L., Jamet A., Ledue F., Blottiere H.M., Lapaque N. (2018). Butyrate produced by gut commensal bacteria activates TGF-beta1 expression through the transcription factor SP1 in human intestinal epithelial cells. Sci. Rep..

[87] Tayefi M., Shafiee M., Kazemi-Bajestani S.M.R., Esmaeili H., Darroudi S., Khakpouri S., Mohammadi M., Ghaneifar Z., Azarpajouh M.R., Moohebati M. (2017). Depression and anxiety both associate with serum level of hs-CRP: A gender-stratified analysis in a population-based study. Psychoneuroendocrinology.

[88] Minihane A.M., Vinoy S., Russell W.R., Baka A., Roche H.M., Tuohy K.M., Teeling J.L., Blaak E.E., Fenech M., Vauzour D. (2015). Low-Grade inflammation, diet composition and health: Current research evidence and its translation. Br. J. Nutr..

[89] Alisson-Silva F., Kawanishi K., Varki A. (2016). Human risk of diseases associated with red meat intake: Analysis of current theories and proposed role for metabolic incorporation of a non-human sialic acid. Mol. Asp. Med..

[90] Silva Meneguelli T., Viana Hinkelmann J., Hermsdorff H.H.M., Zulet M.A., Martinez J.A., Bressan J. (2020). Food consumption by degree of processing and cardiometabolic risk: A systematic review. Int. J. Food Sci. Nutr..

